# Transfer of Non-Dioxin-Like Polychlorinated Biphenyls (ndl-PCBs) from Feed and Soil into Hen Eggs

**DOI:** 10.1021/acs.jafc.2c02243

**Published:** 2022-07-15

**Authors:** B. Ohlhoff, D. Savvateeva, J. Leisner, F. Hartmann, K.-H. Südekum, T. Bernsmann, M. Spolders, A. Jahnke, A. Lüth, I. Röhe, J. Numata, R. Pieper

**Affiliations:** † Department Safety in the Food Chain, German Federal Institute for Risk Assessment, Max-Dohrn-Straße 8-10, 10589 Berlin, Germany; ‡ State Office for Nature, Environment and Consumer Protection (LANUV), North Rhine-Westphalia, 45659 Recklinghausen, Germany; § Institute of Animal Science, University of Bonn, 53115 Bonn, Germany; ∥ Chemical and Veterinary Analytical Institute Münsterland-Emscher-Lippe (CVUA-MEL), 48147 Münster, Germany

**Keywords:** feed-to-food transfer, non-dioxin-like polychlorinated biphenyls, persistent organic pollutants, soil contamination, Gallus gallus domesticus

## Abstract

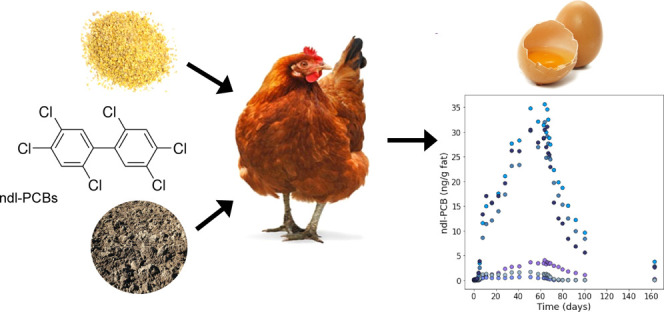

Understanding the transfer of non-dioxin-like polychlorinated biphenyls (ndl-PCBs) into foods of animal origin is crucial for human health risk assessment. In two experiments, we investigated the transfer of ndl-PCBs from contaminated feed and soil into eggs and meat of laying hens. The transfer from the feed was investigated with 30 laying hens. The treated hens were divided into two groups fed a contaminated diet (12.8 μg/kg sum of indicator ndl-PCBs; 88% dry matter (DM)) for 28 and 63 days, respectively, and then experienced a depuration period of 100 days with control feed. The transfer from soil was investigated with 72 laying hens kept in three separate outdoor pens (with three levels of ndl-PCB soil contamination) for 168 days. In both experiments, eggs were collected and analyzed for ndl-PCBs. In the second experiment, animals (*n* = 3 at the beginning, *n* = 6 per group after 42, 84, and 168 days) were slaughtered to determine ndl-PCBs in meat (breast muscle tissue) fat. The transfer of ndl-PCB from both feed and soil was clearly measurable and concentrations in eggs quickly exceeded maximum levels. Clear differences between individual congeners were observed. In particular, the low-chlorinated ndl-PCBs 52 and 101 are hardly found in eggs, despite their relatively high concentration in feed and soil. PCBs 138, 153, and 180, on the other hand, were found in large proportions in eggs and meat.

## Introduction

Polychlorinated biphenyls (PCBs) are a group of persistent organic pollutants that differ in the number and position of the chlorine atoms on the aromatic rings. Each PCB is denoted by a congener number (out of a total of 209) and has distinct toxicological properties. Some congeners have a molecular conformation and toxicological profile similar to polychlorinated dibenzo-p-dioxins and dibenzofurans (PCDD/Fs) and are thus called dioxin-like PCBs (dl-PCBs). All other PCBs do not exhibit dioxin-like properties, have a different toxicological profile, and are therefore referred to as non-dioxin-like PCBs (ndl-PCBs).^1^ In studies with laboratory animals exposed to ndl-PCBs, adverse effects included thyroid, liver, and brain biochemistry, as well as estrogenic, immunotoxic, neurodevelopmental, and reproductive effects.^2^ As ndl-PCBs often occur as mixtures and are often accompanied by PCDD/Fs and dl-PCBs, the establishment of congener-specific toxicological profiles for ndl-PCBs is notoriously difficult. For congener PCB 153, which occurs most frequently in human tissues, a damaging effect on the liver, thyroid, and reproductive organs was demonstrated in a long-term experiment on rats. Several of the indicator PCBs have in vitro and in vivo studies pointing to hepatotoxicity, thyroid toxicity, and neurodevelopmental or neurotoxic effects. The least well-understood toxicological profiles are those for PCBs 101 and 138.^3^ The sum of six indicator ndl-PCB congeners (PCBs 28, 52, 101, 138, 153, and 180) is often reported as a convenient marker for ndl-PCBs and human exposure, as they collectively account for about half of the total ndl-PCBs being present in feed and food. For these indicator PCBs, regulations in the European Union specify the content in food and feed to minimize the risk for humans.^4−6^ Due to their chemical properties such as fat solubility, ndl-PCBs can accumulate in edible tissues of farm animals and can also be transferred into, e.g., milk and eggs.^7,8^ Thus, it seems logical that a (positive) linear relationship has been found, e.g., between the levels in feed and hen eggs.^7,9^ However, the quantitative congener-specific behavior of ndl-PCB accumulation and excretion in laying hens has so far received little attention.

Due to their persistence, ndl-PCBs are present ubiquitously in the environment (e.g., air, soil, sediments) with soils and sediments acting as primary reservoirs. In addition to contaminated feed, the ingestion of contaminated soil particles by chickens while foraging is considered the main cause of egg contamination.^7,10,11^ To assess the animal exposure, not only the concentration of PCBs in the soil should be considered but also the amount of ingested soil particles, which may be affected by stocking densities and feeding regimes.^12,13^

Here, we present two experiments and one case study with laying hens to evaluate the effects of contaminated diet and soil as sources of ndl-PCBs and the respective congener-specific accumulation in tissues and excretion via eggs.

## Materials and Methods (Including Safety Information)

### Experiment 1: Laying Hens Feeding Study with ndl-PCB-Contaminated Feed

#### Ethics Approval Statement

All experimental procedures involving animals were registered by the Regional Office for Health and Social Affairs (LAGeSo) in Berlin, Germany, under StN006/19.

#### Animal Husbandry, Study Design, and Experimental Diets

Laying hens (white leghorn), aged 20 weeks, were kept in two groups (15 hens per group) in pens (14.34 m^2^ per pen) with concrete floor littered with sawdust. The room temperature was maintained at 20 ± 3 °C. The lighting program started at 6 a.m. with 12 h of light per day for the entire experimental period. Feed in mash form and water were provided *ad libitum*. During an adaptation period of 21 days, hens of both groups received a commercial complete compound feed (based on wheat grain, corn grain, soybean meal, rapeseed meal, vegetable oil, and a vitamin and mineral supplement as main ingredients; control diet, Supporting Table 1). Afterward, one group of chickens received a diet contaminated with ndl-PCBs (contaminated diet) for 28 days followed by a 100-day depuration period in which hens were again fed with the control diet (short-term PCB-fed chickens = S-group). The other group of chickens were also fed the ndl-PCB-contaminated diet after the adaptation phase, but for a longer period of 63 days (long-term PCB-fed chickens = L-group) followed by a 100-day depuration period. Concentrations of ndl-PCB congeners in the control diet, PCB diet, and sawdust litter are given in Table 1. The contaminated feed material was obtained from a contamination incident in Germany in 2018. This contaminated feed led to an exceedance of ndl-PCB above the European maximum level of 40 ng/g ndl-PCBs (sum of six indicator congeners) in individual samples of hen egg and chicken meat in different farms in Germany.^5,6^ The origin of this incident was chipped paint, which carried over into the feed from the loading cells of a feed manufacturer. Due to the pneumatic conveying of the feed, the original chipped paint in the feed was converted into evenly distributed fine particles.

**Table 1:** Sum and Individual Concentrations of Six ndl-PCB Congeners (μg/kg ndl-PCBs; 88% Dry Matter (DM))^a^ in Experimental Diets, Littering Material in Exp. 1, and Case Study

	sum^b^	PCB 28	PCB 52	PCB 101	PCB 138	PCB 153	PCB 180
control diet	0.17	0.01	0.05	0.03	0.03	0.03	0.02
contaminated diet	12.77	0.23	0.94	1.46	3.31	4.28	2.55
litter	0.10	0.02	0.02	0.02	0.01	0.02	0.01
case diet	27.00	1.12	2.60	4.23	5.30	7.50	6.25

a Expanded uncertainty of the measurement is 37.7%.

b Sum concentration of six ndl-PCB congeners (PCBs 28, 52, 101, 138, 153, and 180).

#### Sampling

Over the entire course of the experiment, feed intake and laying performance was determined for each group on a daily basis. On certain days, the eggs of each group were collected, numbered, weighed, and separated into egg albumen and egg yolk to determine the ndl-PCB concentration in the egg yolk. Only the fat-rich egg yolk was analyzed because PCBs are lipophilic and are not expected to significantly transfer into the water-rich albumen.^14^ Egg samples from the S-group were collected on the following days: 1, 2, 3, 4, 5, 8, 11, 16, 22, 28, 29, 30, 31, 32, 33, 34, 36, 41, 45, 51, 58, 66, and 128. Egg samples of L-group were collected on the following days: 1, 2, 3, 4, 5, 8, 11, 16, 22, 28, 34, 41, 51, 58, 63, 64, 65, 66, 67, 68, 69, 72, 76, 80, 85, 92, 100, and 163. To determine the background concentration of ndl-PCBs in the eggs, egg samples of both groups were also analyzed prior to the beginning of the experiment, which is defined as day 0.

### Case Study

The feed contaminated with ndl-PCBs for this case study (“case diet”) also originates from the same event in autumn 2018 as experiment 1. This was also attributed to chipped paint carried over into the feed, but likely from a different loading cell than the “contaminated diet” of the same feed manufacturer. For this reason, the concentration of ndl-PCBs and the congener profile of the case diet differ from the corresponding values in the contaminated diet of experiment 1. During further investigation, one small laying hen farm was selected for a follow-up “case study,” where the contaminated case diet caused a very high initial ndl-PCB contamination in hen eggs. Neither the exact feed intake nor the duration of the exposure were recorded. After a switch from case diet to a commercial compound feed, egg samples (i.e., pools of 30 eggs per sample) were analyzed for ndl-PCB concentration. Samples were therefore taken at regular intervals after the end of feeding with the case diet between days 35 and 147.

### Experiment 2: Laying Hens Feeding Study with Contaminated Soil

#### Ethics Approval Statement

All experimental procedures involving animals were authorized by the State Office for Nature, Environment and Consumer Protection (LANUV), North Rhine-Westphalia in Recklinghausen, Germany, with approval Reg #84-02.04.2016.A109.

#### Animal Husbandry and Study Design

Animal experiments were initiated and carried out by LANUV NRW, Germany.^15^ In total, 72 laying hens (Tetra breed), aged 20 weeks, were allocated to three different outdoor areas (24 hens/area), which were separated by 1.80 m high fences and covered with net protections. Each outdoor area (150 m^2^) had a dense grass cover and was equipped with a barn provided with laying nests. Chickens remained in the barns from 5 pm to 8 am the next day. From 8 am to 5 pm, the chickens were able to use both the barns and the outdoor areas. Hens had *ad libitum* access to feed and water and received a commercial complete feed in mash form via feed troughs. The feed had a low background concentration of ndl-PCB of 0.30±0.14 μg/kg DM. Soils of the outdoor areas differed regarding their ndl-PCB concentrations: Soil-LOW showed the lowest concentration of ndl-PCB, Soil-HIGH represented the highest ndl-PCB concentration, and Soil-MID had a ndl-PCB concentration that ranged between that of Soil-LOW and Soil-HIGH (Table 2). The soils were historically contaminated due to their proximity to urban areas known to have some contamination with PCDD/Fs, dl-PCBs, and ndl-PCBs. However, the concentrations of PCDD/Fs and dl-PCBs will not be discussed further as the focus here is on ndl-PCBs. In total, laying hens were kept on the contaminated areas for a period of 168 days.

**Table 2:** Concentrations of ndl-PCB Congeners (μg/kg 88% DM) in Different Soil Variants

	sum of 6 ndl-PCBs	PCB 28	PCB 52	PCB 101	PCB 138	PCB 153	PCB 180
soil-LOW	6.5	0.09	0.10	0.68	2.15	2.16	1.36
soil-MID	8.4	0.12	0.23	1.00	3.10	2.40	1.50
soil-HIGH	9.11	0.09	0.14	1.06	2.98	3.02	1.82

#### Sampling

At experimental days 14, 28, 42, 56, 70, 84, 126, and 168, three eggs per experimental group were collected, numbered, and separated into egg albumen and egg yolk to determine the ndl-PCB concentration in the egg yolk. Additionally, prior to the start of the experiment, defined as day 0, three eggs per group were pooled and analyzed to detect the background ndl-PCB concentration. Furthermore, prior to the start of the experiment (day 0), three chickens were euthanized by barbiturate pentobarbital injection and breast muscles analyzed for background ndl-PCB concentration. At days 42, 84, and 168, six chickens per group were killed and meat samples (breast muscle tissue) were taken to determine the ndl-PCB concentration.

### Analyses of Samples

Concentrations of ndl-PCB were measured in pooled and individual homogenized egg yolk samples of the respective groups. Analyses of ndl-PCB in feed, litter, and in the individual egg yolk samples were conducted by the German National Reference Laboratory (NRL) for Halogenated Persistent Organic Pollutants in Food and Feed located at the German Federal Institute for Risk Assessment (Berlin, Germany). The ndl-PCB concentrations in pooled egg yolk and meat (muscle tissue) were determined by the Chemical and Veterinary Analytical Institute Münsterland-Emscher-Lippe (CVUA-MEL, Münster, Germany). The analysis of feed, litter, and meat (muscle tissue) is described by Ohlhoff et al.^16^ Both laboratories carry out the analysis according to the requirements in Regulation (EU) 2017/644 and are accredited according to DIN EN ISO/IEC 17025. Additionally, both laboratories participate successfully in the same proficiency tests for the determination of dioxins and PCBs in various food and feed matrices of the European Reference Laboratory (EURL). The ndl-PCB concentration in soil was determined by the “dioxin laboratory” of LANUV (State Office for Nature, Environment and Consumer Protection, North Rhine-Westphalia). Analyses were carried out by a special extraction/cleanup method followed by gas chromatography/mass spectrometry, as described by Klees et al.^17^

For PCB analysis in an individual egg yolk, samples were freeze-dried followed by a homogenization step with liquid nitrogen using a cryomill (PULVERISETTE 0, Fritsch, Germany). An Aliquot of 5 g was mixed with anhydrous sodium sulfate and transferred to a glass column. The fat extraction was performed at room temperature using 250 mL of cyclohexane/dichloromethane (1:1). The obtained extract was vacuum evaporated (Büchi, Germany) and the remaining fat was further dried at 70 °C. The extractable lipid content was determined gravimetrically.

The following sample purification step was performed with the MIURA GO-xHT system (MIURA CO., Ltd., Japan) using four different columns (i.e., silica gel impregnated with silver nitrate, silica gel impregnated with sulfuric acid, activated carbon, and alumina). The extracted lipid content was dissolved in 5 mL of hexane, transferred to the first column, and automatically eluted with 95 mL of hexane. The ndl-PCB fraction is caught on the alumina column. This column was then eluted with 2.2 mL of toluene and subsequently concentrated under a nitrogen stream to a final volume of 20 μL.

For PCB analysis in polled egg yolk, samples were thawed and homogenized. An aliquot of 20 g was ground with glass powder and sodium sulfate in a mortar. The free-flowing powder was placed in a glass column and the fat was extracted with a mixture of cyclohexane/dichloromethane (1:1). The extractable lipid content was determined gravimetrically as described for the individual egg yolk. The following cleanup of the samples was performed fully automatically on an LCTech dioxin sample preparation system (DEXTech plus) within 60 min. The cleanup system involved a silica gel column coated with sulfuric acid to destroy the fat matrix, an alumina column to separate dioxins and PCB from interfering matrices, and a carbon column to partition planar from nonplanar compounds and to isolate the non-ortho PCBs from mono-ortho and di-ortho PCBs.

In this method, the sample was loaded in *n*-hexane over the acidic silica column onto the alumina column. The ndl-PCBs were flushed from the alumina column by a mixture of *n*-hexane and dichloromethane (1:1) onto the carbon column with ndl-PCBs being collected as fraction 1. In both laboratories, the stable-isotope labeled analogues of all quantified PCBs were added before the extraction step. Additionally, a ^13^C-labeled PCB recovery standard was added to the sample prior to the measurement. In both laboratories, measurements of the samples were performed by gas chromatography (GC) (Thermo Fisher Scientific) and high-resolution mass spectrometry (HRMS) (DFS, Thermo Fisher Scientific; resolution 10 000; injection of 1 μL). For the determination of the ndl-PCBs, a HT8-PCB 60 m × 0.25 mm × 0.25 μm (SGE Analytical Science Europe Ltd.) column was used. For quality assurance, an internal reference material from a proficiency test and a blank sample were analyzed in the same way as the samples in each analytical series.

The expanded uncertainty was calculated in accordance with the guidance document on measurement uncertainty for laboratories performing PCDD/F and PCB analyses using isotope dilution mass spectrometry from the combination of the uncertainty component describing the random variations with the uncertainty component describing the method and laboratory bias using a coverage factor *k* of 2, which determined a level of confidence of about 95%. For calculation of the uncertainty describing the random variations, a QC sample or a reference material was used. The method and laboratory bias were calculated from proficiency test results. The expanded uncertainty for egg yolk was 34%.

### Statistical Analyses

To define the relationship between variables, Spearman correlation coefficients were computed between the concentrations of ndl-PCBs in egg yolk and meat (muscle tissue) (both expressed per gram of fat) using Python (Python Software Foundation, Python Language Reference, version 3.8.5) library SciPy and Matplotlib. P-values lower than 0.05 were considered statistically significant. All analysis results are presented as mean values ± standard deviation (SD). The visual representation of the data was generated with Python library NumPy, Pandas, and Plotly.

## Results and Discussion

### Experiment 1: Laying Hens Feeding Study with ndl-PCB-Contaminated Feed

In experiment 1, the laying performance of chickens was affected by variations in the duration of feeding the ndl-PCB-contaminated feed. From day 1 to day 28, the performance of chickens of both experimental groups was comparable. However, from day 29 to day 128, S-group hens had a higher feed intake and consequently a higher egg production compared to L-group hens. Due to the higher egg production, S-group hens also displayed a superior feed efficiency compared to L-group hens (Table 3). The lower laying performance of L-group hens could be a result of the ndl-PCB-contaminated feed because PCBs in the feed can have a negative impact on feed consumption and egg production of laying hens.^18,19^ Recent studies, however, found no effect of ndl-PCB-contaminated feed on the performance of the chickens, but these studies were conducted with much shorter feeding periods (7 and 14 days, respectively).^20,21^ Nevertheless, the influence of dietary PCBs on the laying performance may vary depending on PCB congeners and PCB doses as well as the duration of PCB intake.

**Table 3:** Average Daily Performance of Laying Hens Fed with Different Experimental Diets during the Exposure Phase (day 1 to day 28 for S-Group^a^; day 1 to day 63 for L-Group^b^) and Depuration Phase (day 29 to day 128 for S-Group; day 64 to day 163 for L-Group)^c^

	days 1–28		days 29–63		days 64–128		days 129–163	
	S-group^a^	L-group^b^	S-group^a^	L-group^b^	S-group^a^	L-group^b^	S-group^a^	L-group^b^
feed intake (g/hen)	86.3 ± 14.8	78.8 ± 13.2	108.6 ± 7.8	74.6 ± 12.8	121.2 ± 12.9	118.1 ± 20.6		124.3 ± 8.4
egg production (%)^d^	79 ± 13	78 ± 24	69 ± 13	49 ± 15	95 ± 19	75 ± 26		66 ± 15
egg mass (g)	54.6 ± 2.0	52.4 ± 1.7	57.4 ± 1.0	56.2 ± 1.3	59.4 ± 1.2	58.2 ± 1.8		59.1 ± 2.2
yolk mass (g)	13.4 ± 0.6	13.5 ± 0.4	15.0 ± 0.8	15.3 ± 0.7	17.2 ± 0.9	16.2 ± 0.6		17.1 ± 1.4
feed efficiency (g feed/g egg mass)	2.1 ± 0.5	2.3 ± 0.9	2.2 ± 0.4	3.0 ± 1.6	2.2 ± 0.6	3.0 ± 1.0		3.5 ± 0.9

a S-group = short-term PCB-fed chickens receiving the ndl-PCB-contaminated diet for 28 days.

b L-group = long-term PCB-fed chickens receiving the ndl-PCB-contaminated diet for 63 days.

c Data are means ± SD of 15 chickens per group.

d Egg production (%) = (number of eggs laid/number of animals) × 100.

**Figure 1:**
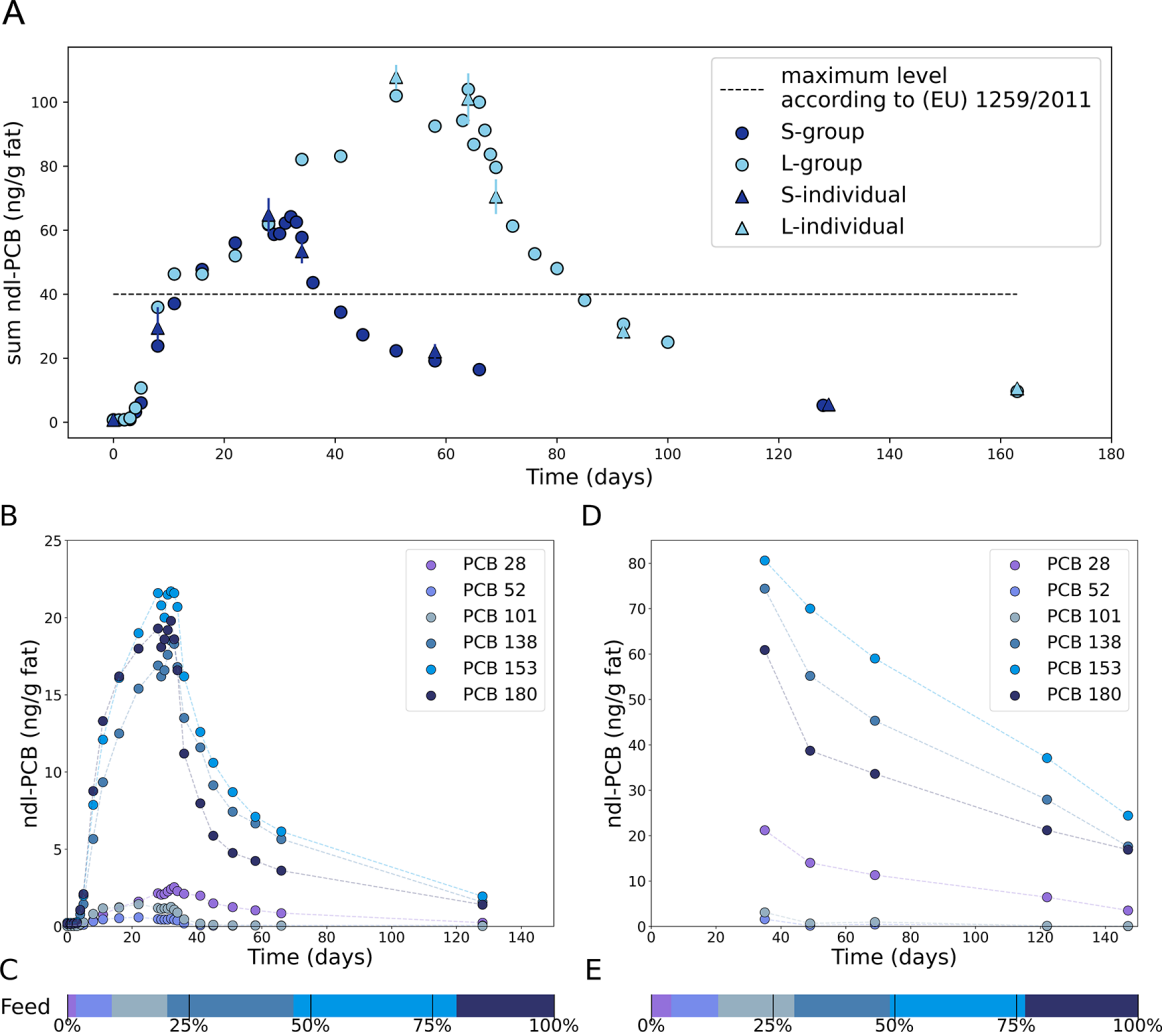
(A–C) Experiment 1; (D, E) case study. (A) ndl-PCB concentrations in egg yolk (ng PCB6/g fat) of short-term (S-group) and long-term (L-group) PCB-fed laying hens as a function of time of the feeding experiment. Dots represent the ndl-PCB concentration of a pooled egg sample of laid eggs per group and day. Triangles represent the mean ndl-PCB concentration of three individual nonpooled single-egg samples per group and day (S/L-individual). The dashed line shows the current EU maximum levels for the sum of six indicator ndl-PCBs for foods of animal origin according to Regulation (EU) 1881/2006 last amended by 1259/2011.^5,6^ (B) Concentration (ng/g fat) of the individual six indicator ndl-PCB congeners in egg yolk of S-group hens and (D) egg yolk of case-study hens. (C, E) Individual congener profiles of six indicator ndl-PCBs in feed used in experiment 1 (12.77 μg/kg sum of six ndl-PCBs; 88% DM) and the case study (27.00 μg/kg sum of six ndl-PCBs; 88% DM).

The concentration of ndl-PCBs increased rapidly after feeding of the contaminated diet in both groups in experiment 1 (Figure 1A). Hens in both groups showed a similar egg yolk ndl-PCB concentration during this period. Hens in the L-group exceeded the EU maximum level of 40 ng/g ndl-PCB^5^ in the eggs already after 11 days, whereas the S-group exceeded these limits after 16 days. The highest concentration in the yolk was measured on day 32 (64.2 ± 12.8 ng/g fat ndl-PCB), which was day 4 after returning to control diet. The ndl-PCB levels further decreased by 95% to 5.3 ± 1.1 ng/g fat ndl-PCB in the yolk after 100 days of depuration (feeding with the control diet). The L-group showed the highest concentration in egg yolk of 104.0 ± 20.8 ng/g fat ndl-PCBs after 51 days of feeding with the contaminated diet. Again, depuration resulted in decreasing ndl-PCB concentration in the eggs. After 100 days, concentrations decreased by 91% to 9.7 ± 2.0 ng/g fat ndl-PCB in the yolk. The highest concentration in the yolk was 64.2 ± 12.8 ng/g fat ndl-PCB and decreased by 95% to 5.3 ± 1.1 ng/g fat ndl-PCB in the yolk after 100 days of depuration (feeding with the control diet).

In experiment 1, the maximum levels according to Regulation (EU) No. 277/2012 for the complete feed of 10 μg/kg (88% DM) ndl-PCBs (sum of six indicator congeners) were only slightly exceeded, with feed levels of 12.77 ± 4.81 μg/kg ndl-PCBs (88% DM).^4^ However, the resulting concentrations of ndl-PCBs in egg yolk were more than 2.5 times the maximum level of 40 ng/g fat ndl-PCB (sum of six indicator congeners) in eggs according to Regulation (EU) 1881/2006 last amended for the ndl-PCBs by (EU) 1259/2011.^5,6^ This reinforces that the transfer characteristics should be taken into account when setting the limits.^22^ Transfer here is defined as the transfer of the parent compound (an ndl-PCB) from feed or soil unchanged into food, since the experiment did not measure the metabolites. Regarding possible exposure routes other than feed, the bedding material (litter) had a detectable but very low background contamination (0.10 ± 0.04 μg/kg ndl-PCB 88% DM; Table 1). At this low concentration, even a very high intake of 50 g/days at 100% absorption of ndl-PCBs would cause negligible differences.^16^

In the L-group, no clear steady state for ndl-PCB concentration in eggs was reached where the contaminated diets were fed for 63 days. This was also seen in other studies with higher concentrations of ndl-PCB in the diet.^7,23^ Interestingly, there was a short increase in ndl-PCB concentration in eggs even during the depuration period (i.e., shortly after the feed change from the contaminated diet to the control diet), followed by a rapid decrease. This is attributable to the gradual production of each egg yolk inside the laying hen, which takes up to 12 days,^24^ so that the new diet determines the transfer to the forming yolks with a delay.^7,20^

Individual congener patterns in feed and (maybe) food of animal origin may help to identify the source of the contamination.^25^ However, individual PCB congeners are partly accumulated, metabolized, and/or eliminated to different degrees in the tissue. This may explain different concentrations in eggs in the present study (Figure 1B). In addition, the ratio of ndl-PCB congeners in feed and eggs differed, which further reinforces a congener-specific transfer from feed to eggs. On the other hand, the ratio of the individual congeners in eggs did not differ between the S- and L-groups (data not shown). The PCB congeners revealed an enrichment of PCBs 28, 138, 153, and 180 in the yolk, whereas PCBs 52 and 101 showed a lower transfer. Similar patterns were observed in eggs of the laying hen farm during the depuration period after the feed contamination incidence (Figure 1C). Although not yet clarified, this could be either related to the relatively lower concentration of PCBs 28, 52, and 101 in the feed (Figure 1D,E) or could also be a result of chlorination degree of ndl-PCB congeners. However, PCB 28 had the lowest concentration in the feed, and the concentration in eggs was higher as compared with PCBs 52 and 101. Similar observations regarding congener patterns were previously reported for laying hens^7,26^ and broilers^16,27,28^ and could be related to previous observations showing that congeners with a lower degree of chlorination are more readily metabolized than more highly chlorinated congeners. Furthermore, chlorination at the *para* position of a biphenyl ring interferes with its metabolization.^29^ As suspected previously, the transfer rates to the egg appeared to be selective for the bioaccumulative and persistent PCB congeners in meat, namely, PCBs 138, 153, and 180.^30^ Egg yolk consists of 32% lipids.^31^ Hence, persistent and bioaccumulative congeners and compounds are more likely to be transferred to the eggs along with the lipids.^32^ The PCBs with higher transfer rate (PCBs 28, 138, 153, 180) were slowly eliminated as these congeners are found more frequently in the egg. In turn, previous studies have also shown that PCBs 52 and 101 are eliminated rather fast.^27^ Most ndl-PCBs are expected to have 80–100% absorption from feed.^16,33^ Combined with the experimental data that shows very low unmetabolized ndl-PCBs in chicken excreta below 1% of the mass balance for all congeners,^34^ we conclude that substances not detected either in eggs or in fat were in fact mostly metabolized.

### Experiment 2: Laying Hens Feeding Study with Contaminated Soil

Laying performance was ∼90% in all three barns over the entire experimental period, with a slightly decreasing tendency in the period between November and January (months 4–6 of the experimental period), likely due to lower outside temperatures. Feed consumption was not recorded (*ad libitum* access to feed) and was assumed to be between 120 and 130 g/day per chicken.

**Figure 2:**
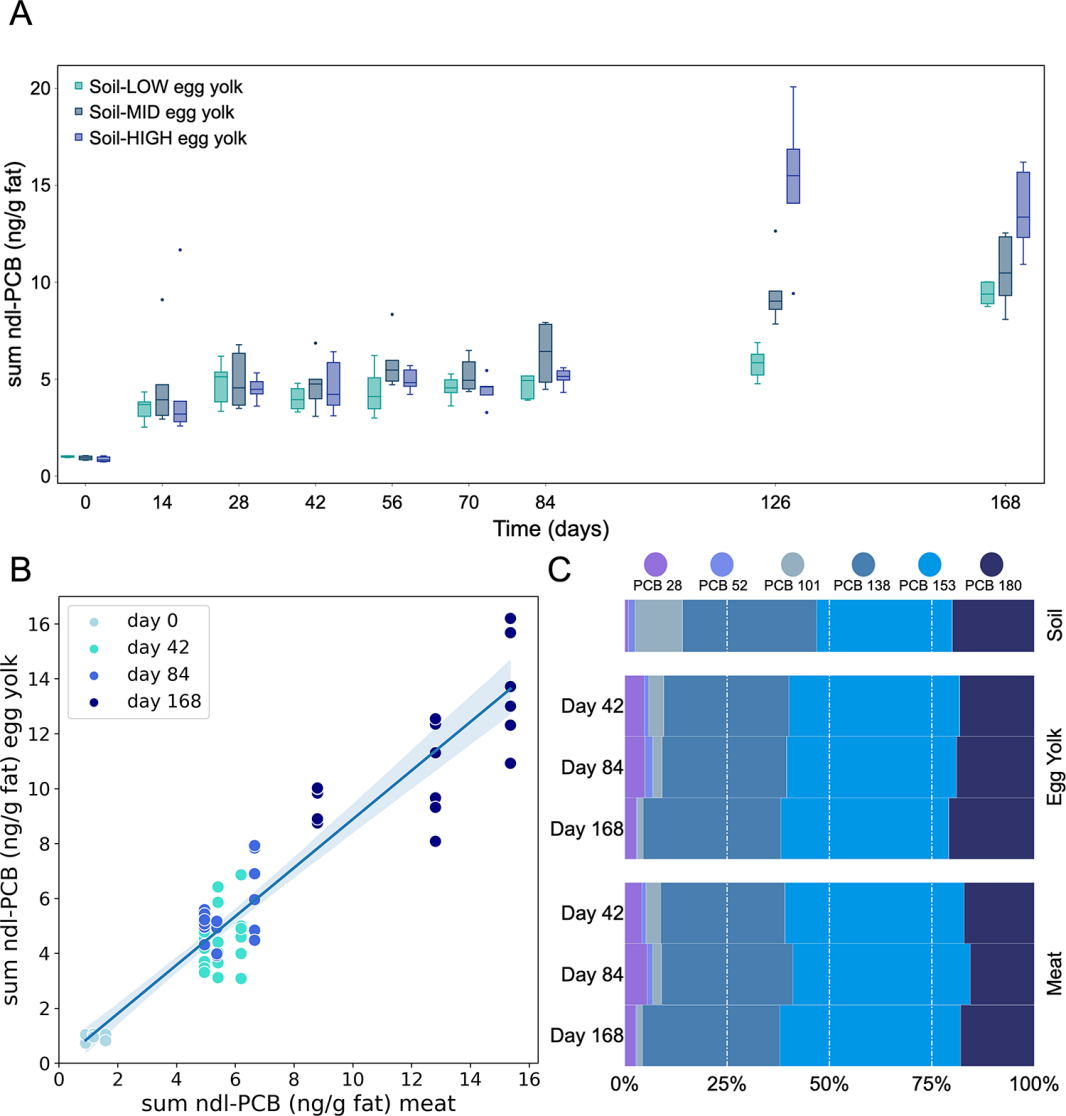
Experiment 2: (A) Sum of six ndl-PCB concentrations (in ng/g fat) in the egg yolk as a function of experimental time (days) for the three different soil levels. (B) ndl-PCB concentration in chicken meat (*x*-axis) against the concentration in the egg yolk (*y*-axis), both expressed per gram of fat, differentiated by the test days. In each case, six egg samples are plotted against one meat sample. (C) Example for congener profile of soil, feed, egg yolk, and meat (hens kept on soil-HIGH).

The ndl-PCB soil contents of Soil-LOW, -MID, and -HIGH were of a similar order of magnitude and did not show large variability. Free-range laying hens likely ingest soil particles during natural feeding behavior, and thus, soil contamination levels should be taken into account regarding their possible transfer into hen eggs. They also consume insects, worms, and herbs together with soil, and that this may be an important part of the consumed contaminants.^35^ In this study, we only measure the soil itself and present it as one of the two sources (feed and soil). In this sense, the soil ndl-PCBs are a proxy for the ndl-PCBs consumed also from the worms and insects (and herbs) therein. It has been reported that free-range chickens can consume up to 30 g of soil per day,^12,36^ which would result in ndl-PCB uptake between 200 and 300 ng ndl-PCB per hen per day in the present study. Considering the mean intestinal absorption for ndl-PCB from contaminated soil between 40 and 60%,^7,33^ it becomes comprehensible that ndl-PCB levels in egg yolk and meat fat increased rapidly in all three groups (Table S2 and Figure 2A). The small differences in ndl-PCB concentration (Soil-LOW: 6.5 μg/kg ndl-PCBs, Soil-MID: 8.4 μg/kg ndl-PCB, Soil-HIGH: 9.1 μg/kg) did not lead to significant differences between groups. In all soil variants, ndl-PCB levels in egg yolk increased sharply immediately after the start of the experiment and then further increased gradually until day 84 after which they again showed a sharp increase. This observation partly confirms previous observations in transfer studies and models showing a biphasic increase in egg levels: first a rapid increase in the initial phase shortly after contamination, followed by a phase of slow increase until steady state.^7,33^ Surprisingly, a strong increase in egg ndl-PCB concentration occurred in all groups on experimental days 126 and 168 (Figure 2A). The reasons are not totally clear since feed and housing materials were not changed. Soil uptake by free-range laying hens depends on many factors including time spent outside or access to feed.^37,38^ Thus, it could be most likely explained by differences in the foraging behavior and soil uptake during the season between November and January (fall, winter) where vegetation cover and plant biomass in outdoor range areas declines. Factors such as the area covered with soil and the animal density also play an important role in soil absorption. In experiment 2, the chickens had about 6–7 m^2^ per chicken, which is even more than in the organic farming systems.^39^

Concomitant to egg ndl-PCB concentration, a similar increase in chicken meat (muscle tissue expressed on fat basis) was observed over the experimental period (Table 4).

**Table 4:** Concentrations of the Sum of Six ndl-PCB (ng/g Fat) in Meat of Chicken Kept on Three Different soils^a^

	ndl-PCB contents (ng PCB6/g fat)		
time (days)	soil-LOW^b^	soil-MID^c^	soil-HIGH^d^
0	1.4 ± 0.6	1.6 ± 0.7	0.9 ± 0.4
42	5.0 ± 1.7	6.2 ± 3.2	5.4 ± 3.3
84	5.4 ± 1.7	6.7 ± 2.3	5.0 ± 1.7
168	8.8 ± 1.6	12.8 ± 2.7	15.4 ± 4.3

a Data are mean ± SD of ndl-PCB concentrations of three meat (muscle tissue in fat basis) samples per group per day.

b Soil-LOW: 6.5 μg/kg ndl-PCBs; 88% DM.

c Soil-MID: 8.4 μg/kg ndl-PCBs; 88% DM.

d Soil-HIGH: 9.11 μg/kg ndl-PCBs; 88% DM.

There was a linear relationship between the ndl-PCB concentration in meat fat and the concentration in eggs (Figure 2B). The slope of Figure 2B was fitted as 0.89, indicating that the concentration in egg yolk was slightly lower than in meat, which may be due to dilution effects during rather rapid egg yolk production compared to body fat accumulation during the laying period in hens.^24^ At the level of individual ndl-PCB congeners, a significant correlation was also observed between eggs and meat, ranging from 0.83 to 0.91 (data not shown).

The meat also did not exceed the EU maximum level.^5^ Although the liver was not measured, we know from our previous study on fattening chickens^16^ that compliant meat ensures that the liver will also be compliant with the respective EU maximum level.^40^ The congener profiles of PCDD/F or PCB-contaminated feed and food may provide information about the initial source of contamination.^25^ In the present study, the ratio of the individual congeners in soil did not differ between the Soil-LOW, -MID, and -HIGH (Table 2). However, differences were observed between soil and eggs (Figure 2C). The congener profiles in yolk and meat are the result of cumulative congener transfer, mostly from soil but also from feed. No clear differences were observed for PCBs 52, 138, 153, and 180. For all congeners, the profiles for egg and meat were very similar. In eggs, PCB 28 was present at higher proportions compared to soil, whereas the opposite was true for PCB 101. There is an increase in PCBs 28 and 52 between days 42 and 84, followed by a decrease between days 84 and 168, which cannot be easily explained. This could be due to volatility and overlooked background contamination sources. This was also observed for PCBs 28 and 52 in an upcoming manuscript on PCDD/F and PCB feed to cow’s milk transfer study.^41^ For this reason, the data on PCBs 28 and 52 from Experiment 2 should be put into question. This suggests differences in either absorption, metabolism, or excretion of individual congeners in laying hens. The relatively low concentration of lower chlorinated PCBs 52 and 101 in eggs in both experiments presented here are in line with previous studies in laying hens^7,26^ and fattening chickens.^16,27,28^ Previous studies have shown that the chlorination of the individual congeners in soil can have an influence on absorption from the digestive tract.^7,21^ In addition, the vicinal hydrogen atoms in the *meta/para* position may have an effect on clearance time from the body.^20,27^ However, laying hens in experiment 2 had continuous access to contaminated soil, whereas, e.g., laying hens in experiment 1 were fed with noncontaminated feed during the “depuration period”. This will likely allow to gain deeper insight into the transfer kinetics and elimination behavior by toxicokinetic modeling.^42^

Hen eggs are a sensitive indicator for the presence of ndl-PCB in feed or the environment because relatively low oral intake can result in considerable ndl-PCB concentration in the egg yolk. Although the risk of exceeding the maximum levels in eggs is higher with contaminated feed, the intake of ndl-PCBs through foraging on the ground should be taken into account by free-range housing systems. The present studies provide quantitative insights into the relationship between ndl-PCB levels in feed, soil, and hen eggs and will be used as a basis to establish toxicokinetic models to better predict ndl-PCB transfer into food of animal origin in the future.

## Supporting Information Available

Chemical composition for both control and contaminated diet for experiment 1; concentration of ndl-PCBs in egg yolk of hens on the three different soil variants for experiment 2 (PDF)

## Abbreviations

BfR: German Federal Institute for Risk Assessment, Berlin, Germany
LANUV: State Office for Nature, Environment and Consumer Protection, North Rhine-Westphalia, Recklinghausen, Germany
CVUA-MEL: Chemical and Veterinary Analytical Institute Münsterland-Emscher-Lippe, Münster, Germany
LAGeSo: Regional Office for Health and Social Affairs, Berlin, Germany
DM: dry matter
ndl-PCB: non-dioxin-like polychlorinated biphenyls
PCDD/F: polychlorinated dibenzodioxins (PCDDs) and polychlorinated dibenzofurans (PCDFs)
PCB 28: 2,4,4′-trichlorobiphenyl
PCB 52: 2,5,2′,5′-tetrachlorobiphenyl
PCB 101: 2,4,5,2′,5′-pentachlorobiphenyl
PCB 138: 2,2′,3,4,4′,5′-hexachlorobiphenyl
PCB 153: 2,2′,4,4′,5,5′-hexachlorobiphenyl
PCB 180: 2,2′,3,4,4′,5,5′-heptachlorobiphenyl
S-group: short-term PCB-fed chickens
L-group: long-term PCB-fed chickens
soil-LOW: outdoor area with lowest concentration of ndl-PCBs
soil-MID: outdoor area with a concentration of ndl-PCBs in the middle
soil-HIGH: outdoor area with highest concentration of ndl-PCBs
