# Supplementary material for: Transfer of Non-Dioxin-Like Polychlorinated Biphenyls (ndl-PCBs) from Feed and Soil into Hen Eggs
Source: J Agric Food Chem. 2022 Jul 15;70(29):8955–62. doi: 10.1021/acs.jafc.2c02243 (PMC9336649; doi:10.1021/acs.jafc.2c02243)
Supplement: Supplementary file 1 — jf2c02243_si_001.pdf [file jf2c02243_si_001.pdf]

Supporting information

Transfer of non-dioxin-like polychlorinated biphenyls (ndl-PCBs) from feed and soil into hen eggs

Ohlhoff B.[1], Savvateeva D.[1], Leisner J.[2], Hartmann F.[2], Südekum, K.-H.[3], Bernsmann T.[4], Spolders M.[1], Jahnke A.[1], Lüth A.[1], Lahrssen-Wiederholt M.[1], Röhe I.[1], Numata J.[1], Pieper R.[1*]

[1] *Department Safety in the Food Chain, German Federal Institute for Risk Assessment, Berlin, Germany*

[2] *State Office for Nature, Environment and Consumer Protection (LANUV), North Rhine-Westphalia, Recklinghausen, Germany*

[3] *Institute of Animal Science, University of Bonn, 53115 Bonn, Germany*

[4] *Chemical and Veterinary Analytical Institute Münsterland-Emscher-Lippe (CVUA-MEL), Münster, Germany*

Correspondence to: robert.pieper@bfr.bund.de, German Federal Institute for Risk Assessment, Max-Dohrn-Str. 8-10, 10589 Berlin, Germany

Table S1 - Chemical composition of diets per supplier information[1]

**Analyzed nutrients (%)**	**Control diet**	**ndl-PCB contaminated diet**
Crude protein	16.0	17.5
Crude fat	3.5	6.0
Crude fiber	5.0	4.0
Crude ash	12.5	12.0
Lysine	0.75	0.80
Methionine	0.33	0.40
Calcium	3.5	3.6

Phosphorus	0.5	0.45
Sodium	0.14	0.15
ME (MJ/kg)	11.0	11.5

[1] Feed produced by EQUOVIS GmbH, Münster, Germany (Control diet).

Table S2 - Sum of ndl-PCB in the egg yolk of hens reared on the different soil variants.[1]

Day	Sum of ndl-PCB in egg yolk (in ng PCB6/g fat) of hens kept on		
	Soil-LOW	Soil-MID	Soil-HIGH
0	1.01 ± 0.05	0.93 ± 0.12	0.86 ± 0.16
14	3.52 ± 0.64	4.63 ± 2.30	4.55 ± 3.51
28	4.83 ± 1.06	4.89 ± 1.37	4.50 ± 0.58
42	3.99 ± 0.59	4.74 ± 1.26	4.58 ± 1.29
56	4.33 ± 1.19	5.81 ± 1.33	4.94 ± 0.55
70	4.54 ± 0.58	5.18 ± 0.83	4.45 ± 0.71
84	4.68 ± 0.58	6.32 ± 1.48	5.10 ± 0.45
126	5.81 ± 0.80	9.44 ± 1.67	15.24 ± 3.54
168	9.41 ± 0.61	10.54 ± 1.80	13.64 ± 2.01

[1]Data are means ± SD of ndl-PCB concentrations of three eggs per group and day.
